# Harm reduction for the treatment of patients with severe injection-related infections: description of the Jackson SIRI Team

**DOI:** 10.1080/07853890.2021.1993326

**Published:** 2021-11-02

**Authors:** David P. Serota, Hansel E. Tookes, Belén Hervera, Babley M. Gayle, Cara R. Roeck, Edward Suarez, David W. Forrest, Michael A. Kolber, Tyler S. Bartholomew, Allan E. Rodriguez, Susanne Doblecki-Lewis

**Affiliations:** a Division of Infectious Diseases, Department of Medicine, University of Miami Miller School of Medicine, Miami, FL, USA; b Jackson Memorial Hospital, Jackson Health System, Miami, FL, USA; c Department of Public Health Sciences, University of Miami Miller School of Medicine, Miami, FL, USA

**Keywords:** Endocarditis, substance use disorder, skin and soft tissue infection, opioid use disorder

## Abstract

**Introduction:**

Hospitalizations for severe injection-related infections (SIRI), such as endocarditis, osteomyelitis, and skin and soft tissue infections (SSTI) are increasingly common. People who inject drugs (PWID) experiencing SIRIs often receive inadequate substance use disorder (SUD) treatment and lack of access to harm reduction services. This translates into lengthy hospitalizations with high rates of patient-directed discharge, readmissions, and post-hospitalization mortality. The purpose of this study was to describe the development of an integrated “SIRI Team” and its initial barriers and facilitators to success.

**Materials and methods:**

The Jackson SIRI Team was developed to improve both hospital and patient-level outcomes for individuals hospitalized with SIRIs at Jackson Memorial Hospital, a 1550-bed public hospital in Miami, Florida, United States. The SIRI Team provides integrated infectious disease and SUD treatment across the healthcare system starting from the inpatient setting and continuing for 90-days post-hospital discharge. The team uses a harm reduction approach, provides care coordination, focuses on access to medications for opioid use disorder (MOUD), and utilizes a variety of infection and addiction treatment modalities to suit each individual patient.

**Results:**

Over the initial 8-months of the SIRI Team, 21 patients were treated with 20 surviving until discharge. Infections included osteomyelitis, endocarditis, bacteraemia/fungemia, SSTIs, and septic arthritis. All patients had OUD and 95% used stimulants. All patients were discharged on MOUD and 95% completed their prescribed antibiotic course. At 90-days post-discharge, 25% had been readmitted and 70% reported taking MOUD.

**Conclusions:**

A model of integrated infectious disease and SUD care for the treatment of SIRIs has the potential to improve infection and addiction outcomes. Providing attentive, patient-centered care, using a harm reduction approach can facilitate engagement of this marginalized population with the healthcare system.

**KEY MESSAGES**
- Integrated infectious disease and addiction treatment is a novel approach to treating severe injection-related infections.
- Harm reduction should be applied to treating patients with severe injection-related infections with a goal of facilitating antibiotic completion, remission from substance use disorder, and reducing hospital readmissions.

## Introduction

Increases in hospitalizations for severe injection-related infections (SIRIs) have mirrored the ongoing crisis of drug overdose deaths over the last 10 years [1,2]. However, infections due to injection drug use (IDU) have not garnered the same level of policy or research attention compared to drug overdose deaths [3,4]. In the United States, incidence rates of opioid use disorder (OUD)-associated endocarditis increased by nearly 80% between 2007 and 2017 [5]. Hospitalizations for SIRIs, which include skin and soft tissue infections (SSTI), bacteraemia, endocarditis, osteomyelitis, and septic arthritis, are characterized by long lengths of stay [6], high readmission rates [7], frequent patient-directed discharge (also known as “against medical advice”) [6], and post-discharge mortality [8]. Over the coming decade, by 2030, it is estimated that more than a quarter-million people will die from injection drug use (IDU)-associated endocarditis in the United States [9]. Despite the increasing burden of SIRIs on patients, providers, and healthcare systems, there is a dearth of prospective research exploring how best to manage this complex syndrome. Aside from medical concerns, patients experiencing SIRIs have complicated needs and face significant barriers to care including unstable housing, food insecurity, limited access to healthcare, and stigma—both within and outside the healthcare system.

A few interventions appear to be beneficial in the management of patients with SIRIs. For patients with SIRIs and OUD, initiation of medications for OUD (MOUD)—including buprenorphine or methadone—is associated with reduced length of stay, less patient-directed discharge, improved completion of antibiotics, and reduced readmission [10–13]. For patients leaving the hospital under patient-directed discharge, providing oral antibiotics is associated with reduced readmission compared to no antibiotics [14]. When available, addiction medicine consultation is associated with improved antibiotic completion, initiation of MOUD, and post-discharge treatment engagement [15,16]. Beyond the conclusions that patients with SIRIs should receive antibiotics whether they remain in the hospital or leave early and that patients with OUD should receive life-saving medications for OUD [17], different models of care delivery remain less well-defined. Furthermore, while most studies have evaluated interventions for OUD-associated infections, we have shown that 40% of SIRI hospitalizations in Florida involve stimulant use (including cocaine and amphetamines) and that stimulant use alone accounts for over a quarter of SIRI hospitalizations [18]. While progress has been made in the management of SIRI involving opioid use by using MOUD, polysubstance use is common and no similarly effective medication treatment for stimulant use disorder exists.

Integration of substance use disorder (SUD) and infectious disease care might improve outcomes for both conditions of this rapidly expanding syndemic [19]. We hypothesized that integration of infectious diseases and SUD treatment across the healthcare ecosystem would facilitate complete and harmonized treatment of both conditions compared to the current model of fragmented care and often-absent SUD treatment [20,21]. We previously synthesized the available data on the management of SIRIs and applied this information in the development of the SIRI Team intervention [22]. We organized our approach to treating SIRIs into three main components, including (1) treating the acute infection, (2) treating the underlying SUD, and (3) informing all aspects of care with harm reduction principles [23,24]. Herein we describe the development and early experiences of the Jackson Memorial Hospital (JMH) SIRI Team, an intervention aimed at delivering integrated infectious disease and SUD treatment for individuals hospitalized with severe infections from IDU.

## Materials and methods

### Development of the SIRI Team

#### Quantifying the problem

Previous research has demonstrated the cost burden of SIRIs at JMH, the 1550-bed safety-net hospital for Miami-Dade County, Florida, with one-year healthcare expenditures totalling $11.4 million in 2014 [25]. Since then, anecdotal reports of increasing hospitalizations for SIRIs have been confirmed statewide [26]. In response, our institution opened the doors of Florida’s first legal syringe services program (SSP), the Infectious Disease Elimination Act (IDEA) Miami SSP, in 2016. The opening of IDEA Miami SSP has been associated with a reduction in the number of improperly discarded syringes on the streets and a reduced number of hospitalizations for an opioid overdose at JMH [27,28]. The SIRI Team medical director (D.P.S.) is a physician at the IDEA Miami SSP and helps supervise their student-run free clinic [29]. To understand the current burden of SIRIs at JMH in more recent years, we completed a needs assessment by quantifying the problem. We used a search algorithm to identify SIRI hospitalizations using international classification of disease-10 codes (ICD-10) for both an injectable substance use diagnosis and an infection commonly associated with IDU (Supplementary Appendix 1) [26]. Table 1 includes data on the number and outcomes of SIRIs at JMH between 1 August 2018 and 31 July 2019, the year immediately preceding the development of the Jackson SIRI Team. Overall, 413 hospitalizations met the inclusion criteria. Consistent with state-wide data, over 20% left under patient-directed discharge and nearly half were readmitted within 90-days of discharge. Due to the administrative nature of these data, reasons for readmissions were not available. Hospitalizations were long and costly, with most patients having publicly funded insurance (56%) or uninsured status (36%) [25]. These data provided the impetus for JMH leadership to support the development of the SIRI team.

**Table 1.** Descriptive statistics of patients hospitalized for a severe injection-related infection between August 2018 and July 2019.

	Any	Endocarditis	Osteomyelitis	Bacteraemia/sepsis	Septic Arthritis	SSTI
Total, *n*	413	25	69	176	16	127
Median LOS, days	10	17	8	13	12	7
Median charge, USD	$64,429	$87,546	$51,098	$106,079	$56,762	$45,762
In-hospital mortality, % (*n*)	7% (29)	4% (1)	3% (2)	14% (25)	0% (0)	0% (1)
Patient-directed discharge, % (*n*)	21% (80)	25% (6)	22% (15)	17% (25)	44% (7)	21% (27)
90-day readmission, % (*n*)	46% (175)	58% (14)	58% (39)	50% (75)	6% (1)	37% (46)

LOS: length of stay; SSTI: skin and soft tissue infection; USD: United States dollars.

#### Engaging institutional leaders

We met with the hospital chief executive officer and chief medical officer to present these data as well as our past experiences and successes with providing informal addiction medicine services to patients seen on the infectious diseases consult service. We made the financial argument that the hospitalizations are costly, outcomes are poor, and that patients and providers are unsatisfied with the experience. The objectives of the team were presented to improve patient outcomes and mitigate healthcare costs for the system. For patients with SIRIs, the goal is to deliver comprehensive, integrated, patient-centered care and to cure infectious diseases while fostering recovery from addiction. For the healthcare system, the SIRI Team goals are to expedite safe hospital discharges, reduce hospital costs, and prevent readmissions. Funding of the team includes salary support for the medical director, a nurse practitioner, and a team mobile phone. Otherwise, all services are funded as usual clinical care. The IDEA Miami SSP, which assists with patient navigation for SIRI Team patients, is privately funded. We met with stakeholder groups including hospitalists, case managers, social workers, nursing administration, infectious disease faculty, medical house staff, and local residential addiction treatment facilities. During informational sessions, we provided a brief overview of SIRIs, the idea and function of the SIRI Team, and a summary of the SIRI Team methods.

#### General approach

**Figure 1.**
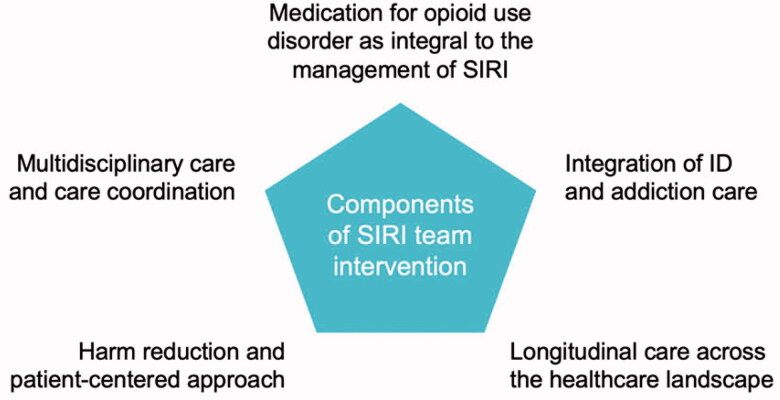
Components of a comprehensive, harm reduction approach to SIRI treatment. ID: infectious diseases; SIRI: severe injection-related infection.

Patients eligible for SIRI Team care included patients hospitalized at JMH with a severe infection associated with IDU and expected need for ≥7 days of antimicrobial therapy. Infections included SSTI, bacteraemia, fungemia, osteoarticular infection, and endocarditis. Figure 1 highlights the guiding principles of the SIRI Team. We sought to alter the paradigm of treating patients with SIRIs from a hospital-based approach to a longitudinal patient-centered relationship that was not bound by the walls of the healthcare system. Care is focussed on respect for the individual and their specific needs. This includes balancing effective infectious disease care with what fits best with each patient’s life circumstances, substance use, and interest in recovery from addiction. A high value is placed on lowering barriers to communication between patients and the SIRI Team by providing all patients with our calling card—a box of naloxone nasal spray with the SIRI Team mobile phone number on it—at the bedside. Although our approach is intensive, we hypothesize that it will result in cost savings by decreasing length of stay (LOS), readmission rate, and improving patients’ recovery from SUD and overall health. The SIRI Team follows patients for ∼90 days post-discharge and works to link to ongoing addiction and infectious disease care, thereafter.

The SIRI Team intervention is informed by harm reduction principles. As evident by the 20% patient-directed discharge rate among patients with SIRI, remaining in the hospital to complete antibiotics is not the ideal treatment plan for many patients [30]. Improving the comfort and experience of hospitalization for patients with SIRI is a high priority for the team, with particular attention to the treatment of pain and substance withdrawal and cravings. For patients who cannot comfortably remain in the hospital, we ensure immediate access to oral antibiotics before leaving the hospital. This includes an antibiotic contingency plan in each SIRI progress note so primary teams know which oral antibiotic option is preferred at the time of patient-directed discharge. Traditionally, when patients leave the hospital on their own terms, they are abandoned by the medical system and receive no follow-up. During initial SIRI Team consultation, patients are informed that the team will continue to care for them regardless of whether they remain in the hospital or leave early, unrestricted by discharge location. We use shared decision-making to determine the most effective and acceptable antibiotic treatment plan, including a variety of discharge locations and antibiotic approaches (Table 2).

**Table 2.** Suite of treatment modalities utilized by the SIRI Team.

Antibiotic strategies	Full course IV antibiotics Induction with IV followed by oral step-down therapy Weekly or every-other-week long-acting IV antibiotic infusion Full course high bioavailability oral antibiotics
SUD treatment strategies	Residential addiction treatment For OUD: Buprenorphine-naloxone, methadone For stimulant use disorder: mirtazapine, topiramate/extended-release, mixed amphetamine salts All: referral to local SSP, provision of naloxone
Location of antibiotic treatment	Hospital Private residence Residential addiction treatment Salvation Army medical respite facility Nursing facility Street with SSP pill locker program
Location of SIRI Team care	Hospital Hospital-based ambulatory care centre Telehealth including videoconference capability Home visit IDEA Miami SSP fixed site and mobile outreach unit

IV: intravenous; OUD: opioid use disorder; SIRI: severe injection-related infection; SUD: substance use disorder; SSP: syringe services program.

Additional harm reduction interventions include preventing and treating other infectious diseases. These interventions are tracked and prompted by using a standardized clinical note template (Supplementary Appendix 2). All patients living with HIV are offered initiation of anti-retroviral therapy (ART), which is managed by the SIRI Team. For patients with chronic hepatitis C (HCV), all baseline labs and studies are obtained to facilitate treatment in the outpatient setting. HCV antivirals are not available on the inpatient formulary. All patients without HIV infection are educated on pre-exposure prophylaxis (PrEP). For those interested, PrEP is prescribed upon hospital discharge or patients are referred to an affiliated low barrier mobile PrEP program.

The SIRI Team provides intensive patient navigation including finding sources of funding for medications, arranging follow-up appointments with specialists, managing home health and infusion services, and delivering medications to patients in the field. Our relationship with the IDEA Miami SSP provides patients experiencing homelessness with a pill locker to store medications safely and access to a substance use-focussed social worker and clinical psychologist, in addition to SSP services. Patients lacking access to the internet or mobile services are invited to stop by the IDEA Miami SSP during business hours to be connected with the SIRI Team by videoconference if there is availability.

Funding for post-hospital medications and specialist follow-ups is provided by private or public insurance for those who have insurance. For the nearly half of SIRI Team patients who are uninsured, medications are covered using safety net funds of the public hospital system. The health system has medical respite beds available for 2–4 week stays post-hospital when patients experiencing homelessness require short-term wound care or home-health nursing service, like intravenous antibiotics. For uninsured patients requiring post-hospital dalbavancin infusions, medication was obtained free of charge using the manufacturer assistance program and infusion centre sessions were paid for with safety net hospital funds.

#### Using implementation science to improve efficacy

Because the SIRI Team remains in the early stages of implementation, rapid-cycle feedback and adjustments to team function are being made regularly. Using the Consolidated Framework for Implementation Research (CFIR), we are conducting a formative evaluation of the SIRI Team to optimize this multicomponent intervention and improve efficacy and reach [31]. This includes conducting qualitative interviews with healthcare providers, health system administrators, and patients with a history of hospitalization for SIRI. A future pre/post-implementation study will be performed to evaluate the efficacy of the SIRI Team on important patient and healthcare system outcomes.

### Statistical analysis

Clinical data were abstracted from medical records. Substance use was determined through a combination of self-report and urine drug screening results. Discharge outcomes are reported in the subset of patients who survived until discharge. Engagement in MOUD was defined as a self-report of taking MOUD at the time of 90-day post-discharge contact. Categorical data are presented as percentages and quantitative data as the median and interquartile range (IQR). Exemplary cases are presented briefly based on SIRI Team experience. This study was approved by the institutional review board of the University of Miami and the clinical trials office of Jackson Memorial Hospital. There was a waiver of informed consent for retrospectively collected data and participants provided informed consent for medical record data collection and publication of quantitative results and case presentations.

## Results

During the initial pilot phase between 1 August 2020 and 11 April 2021, the SIRI Team completed 21 initial consults for patients hospitalized for SIRIs at JMH. Table 3 provides sociodemographic, substance use, and infectious disease information on SIRI Team patients. The median age was 44 years, 38% were female, and half were from a racial or ethnic minority group. Eighty-one percent were experiencing homelessness at the time of hospital admission. All patients used opioids and 90% used cocaine.

**Table 3.** Descriptive statistics of patients seen by the SIRI Team.

Variable*	Percent or median (IQR)
Age	44 (39–48)
Female	38%
Hispanic	48%
Black race	10%
Homeless	81%
Phoneless	52%
Uninsured	48%
HIV	29%
Chronic HCV infection**	52%
Substances used	
Opioids	100%
Cocaine or crack	90%
MDMA	19%
Methamphetamine	14%
Benzodiazepines	14%
Alcohol	5%
Any stimulant	95%
Infections***	
Bacteraemia/fungemia	33%
Endocarditis	14%
Septic arthritis	14%
Non-vertebral osteomyelitis	14%
Vertebral osteomyelitis	14%
Skin and soft tissue infection	33%
Length of stay, days	12 (7–20)
Days of antibiotics	26 (16–42)
Post-discharge antibiotic route (*n* = 20)	
Oral	85%
Intravenous	5%
Long-acting IV	10%
Oral + (intravenous or long-active IV)	10%
None	10%
Completed antibiotic course (*n* = 20)	95%
Discharged on MOUD (*n* = 20)	100%
Patient-directed discharge (*n* = 20)	20%
Discharge location (*n* = 20)	
Home	30%
Street/homelessness	40%
Residential addiction facility	20%
Salvation army medical respite	5%
Nursing home	5%

HCV: hepatitis C virus; HIV: human immunodeficiency virus; IQR: inter-quartile range; IV: intravenous; MDMA: 3,4-methylenedioxy-methamphetamine; SIRI: severe injection-related infection.

*n = 21 unless otherwise stated.

**Chronic HCV infection defined as having a positive HCV antibody and detectable HCV viral load. No patients had suspected acute HCV infection.

***Other infections: orthopaedic hardware infection (1), lung abscess (2).

There was one in-hospital death due to refractory MRSA endocarditis. All but one surviving patient (19/20, 95%) completed their planned antibiotic course, and all were discharged on MOUD (buprenorphine for 19 and methadone for 1). Oral antibiotics were the most common long-term treatment plan post-discharge and two patients completed treatment with infusions of dalbavancin, a long half-life antibiotic dosed weekly through a peripheral IV. Of the four patients who left under patient-directed discharge, all were recommended to receive post-discharge oral antibiotics. Thus, all patients received their first-line antibiotic plan. Five out of 20 (25%) were readmitted within 90-days of discharge. At 90-day post-discharge follow-up, 14/20 (70%) reported continuing to use MOUD. Table 4 presents representative vignettes of patients seen by the SIRI Team, their clinical course, and barriers to their care.

**Table 4.** Select representative case presentations from the SIRI Team.

Case	Description
A	A 49-year-old woman with severe OUD, psychostimulant use, and experiencing homelessness was hospitalized with lumbar vertebral osteomyelitis. She was seen by an initial infectious disease consultant who recommended she remain hospitalized for 6 weeks to receive IV antibiotics. She was seen by the SIRI Team and buprenorphine was initiated. We determined that remaining hospitalized was not beneficial for her recovery and facilitated discharge 3 weeks earlier than planned on oral antibiotics, MOUD, and PrEP. On the day of planned discharge, she became frustrated with staff and left before discharge. The SIRI Team ensured she was still given her medications—including antibiotics, buprenorphine-naloxone, and tenofovir-emtricitabine—before leaving. The patient was followed by telehealth and completed her antibiotics with no complications while staying at a friend’s house.
B	A 48-year-old man with severe OUD, cocaine use disorder, and experiencing homelessness was hospitalized for pathologic fracture of the tibia due to osteomyelitis. The initial infectious disease consult team suggested he remain hospitalized for 6 weeks to receive IV antibiotics. He was seen by the SIRI team, initiated on buprenorphine, and discharged to a residential addiction treatment facility 4 weeks earlier than planned to complete oral antibiotics. At the facility, his buprenorphine was tapered without his consent or consultation with the SIRI Team. Follow-up was completed by videoconferencing. He was transitioned to a sober living facility and the buprenorphine dose was increased back to the treatment dose. Follow-ups were completed by home visits to his front porch. He remains housed and in recovery for the longest period in the last 10 years.
C	A 29-year-old man with severe OUD, cocaine use disorder, and benzodiazepine use disorder was hospitalized for MRSA tricuspid valve endocarditis and orthopaedic hardware infection. He underwent removal of a femoral rod with debridement of infected bone. He was started on buprenorphine by the SIRI Team and completed antibiotics with weekly home infusions of dalbavancin. He continued to use cocaine but has not injected drugs or used opioids since discharge. SIRI Team follow-up was by telephone and a home visit.
D	A 47-year-old man with severe OUD, cocaine use disorder, HIV infection, and experiencing homelessness was hospitalized for infected wounds after being arrested for possession of fentanyl. He was found to have polymicrobial bacteraemia and was seen by the SIRI Team who started him on ART, buprenorphine, and IV antibiotics. The SIRI team coordinated with two doctors in the jail system who ensured he would be continued on buprenorphine and antibiotics if discharged back into their custody. He was released from jail 2 days later with no antibiotics and no medications. Due to a lack of a mobile phone, he was uncontactable. He presented back to the hospital a few days later for re-initiation of his medications and to complete treatment for his infection. SIRI Team again cared for the patient and attempted to get him into residential addiction treatment, per his request; however, no facility would take him stating that his chronic wounds were too complex. Instead, he was discharged to homelessness, this time with medications. He has since been seen in an outpatient HIV clinic and continues to take buprenorphine and ART.

ART: HIV antiretroviral therapy; HIV: human immunodeficiency virus; ID: infectious diseases; IV: intravenous; LOS: length of stay; OUD: opioid use disorder; PrEP: HIV pre-exposure prophylaxis; SIRI: severe injection-related infection.

## Discussion

The Jackson SIRI Team was developed as a response to the epidemic of IDU-associated infectious diseases. Our goal was to improve the care of patients hospitalized with IDU-associated infectious diseases by providing low-barrier, patient-centered, and integrated infectious disease and addiction care. From a health system perspective, we sought to reduce hospital costs and resource utilization while changing institutional culture with regards to people who use drugs. During the initial 8-month pilot phase, 21 patients were evaluated by the team and received integrated infectious diseases and addiction treatment that defied typical inpatient *vs.* outpatient healthcare system silos. All but one patient completed their antibiotics, and all received MOUD during and after hospitalization, with 70% retained in MOUD at 90-days. The SIRI team serves as a new model of care to address the infectious disease/SUD syndemic by focussing on treatment tailored to the individual and informed by harm reduction principles.

The Jackson SIRI Team intervention adds to a growing list of models for integrating infectious disease and addiction care for individuals hospitalized with SIRIs. Two institutions have published their experiences with integrating infectious disease care into residential SUD treatment facilities, post-discharge [32,33]. In both cases, residential treatment was not popular with patients and there were differences between expectations and realities of the patient experience at the facilities. One promising approach is integrating addiction care into post-discharge infectious disease follow-up clinics including the provision of MOUD [34–36]. Fanucchi and colleagues conducted a pilot study of a SIRI Team consisting of MOUD integrated with outpatient intravenous antibiotics. They showed that integrated MOUD and ID care is feasible, associated with shorter lengths of stay, and no worse infection outcomes [12,34]. Although we did schedule patients to follow up in an in-person SIRI clinic, no patient successfully attended an in-person appointment. In our setting with over 80% of patients experiencing homelessness and 50% with phonelessness, the traditional, passive, clinic appointment-based approach to medical care was not feasible. Instead, care was delivered with repeated outreach to patients, calling emergency contacts, visiting patients in the field, the invitation to drop in at our SSP any time, and relaying messages to them by any means necessary.

Compared to other cohorts of patients with SIRI, the 100% rate of post-discharge MOUD is especially notable. National US cohorts have demonstrated <10% receipt of MOUD following SIRI hospitalization [37,38] and even in select locales with higher addiction treatment access, only 25–50% of patients with SIRI receive post-hospital MOUD [10,39]. We believe that the control of cravings and withdrawal facilitated high rates of antibiotic completion in a group of individuals who had previously struggled immensely with adherence to medical care. At the same time, nearly all patients had concomitant stimulant use for which no similarly high-efficacy medication treatments exist. Emerging medication approaches to stimulant use disorder are promising, but efficacy in non-treatment-seeking hospitalized patients, such as those with SIRIs, remains unexplored. Furthermore, while behavioural interventions like contingency management have shown some success in hospitalized patients with stimulant use disorder, such interventions are difficult to operationalize in real-world settings [40].

Despite successes, the SIRI team faced numerous challenges to achieving optimal outcomes for patients. Some health system-level barriers to care included lack of discharge options for people experiencing homelessness, lack of funding for aftercare and medications for uninsured patients, few residential treatment programs willing to accept medically complex patients (Case D), and health system pressure to have patients discharged as soon as possible. Although extraordinary efforts were made to coordinate care across the healthcare system, including acute care hospitals, residential treatment facilities (Case B), homeless shelters, and the jail system, communication and continuity of care are continuing obstacles.

The SIRI Team faced considerable barriers to delivering MOUD to this vulnerable population. Residential addiction treatment facilities regularly tapered buprenorphine without patient or SIRI Team consent (Case B). For insured patients, we were regularly confronted with onerous prior authorization requirements leading to delays in care. Concordant with recent reports from Massachusetts, our experience is that patients with OUD continue to face challenges with placement in skilled nursing facilities and other post-acute care programs [41].

For individual patients, the lack of mobile phones made post-hospital follow-up difficult. Inability to communicate with patients hindered our ability to address gaps in care, replace missing medications (Case D), and stem worsening infections, which ultimately led to rehospitalization for some. Patients often desired to leave the hospital earlier than was advisable, despite attentive management of SUD symptoms. Even in the best-case scenario with SIRI team care, hospitalization is neither a pleasant nor a therapeutic experience for many people who use drugs [30]. The patient directed-discharge rate was similar among SIRI Team patients compared to local historical controls. However, SIRI Team patients who left under patient-directed discharge were immediately contacted and connected with antibiotics, MOUD, and other medications, which has not been a traditional practice in our institution. Unfortunately, in some circumstances, the SIRI Team was unable to anticipate or prevent lapses in treatment, such as for incarcerated patients or those in residential treatment facilities. We are working on how to best balance “patient-centeredness” with the safest high-quality infectious disease treatment as well as how to rapidly adapt treatment plans to non-hospital care sites. A qualitative study funded by our Centre for AIDS Research is currently ongoing to further facilitate the implementation of the SIRI team.

The Jackson SIRI team is supported by a unique care environment that may not be easily adaptable to other health systems. Our close partnership with the IDEA Miami SSP is an indispensable support system to assist with linking to community SUD resources, serving as a trusted institution among the Miami PWID community, and providing low barrier access to wound care, injection supplies, and medication storage. Over the initial 8 months in operation, SIRI Team members have made close connections with key stakeholders within and outside the health system that are crucial to facilitating care for patients. Harm reduction institutions, such as SSPs, serve as an important linkage between the healthcare system and people who use drugs [42]. Expansion of harm reduction programs and access to low barrier evidence-based SUD treatment will be a key facilitator of SIRI Team adoption in other healthcare settings.

## Conclusions

The current approach of fragmented, myopic care for patients with life-threatening infections due to injection drug use is ineffective at fostering recovery and inefficient for healthcare utilization. We demonstrated that the development of an integrated infectious disease/addiction team was feasible and achieved promising patient- and health system-important outcomes. Key characteristics of the SIRI team include a focus on access to MOUD, intensive patient navigation, and longitudinal care indifferent to inpatient/outpatient status. Further study, including a multisite randomized controlled trial, is necessary to facilitate the implementation of the SIRI Team and to formally evaluate the efficacy of this model of integrated infectious disease/addiction care.

## Supplementary Material

Supplemental Material

## Data Availability

Due to the nature of this research, participants of this study did not agree for their data to be shared publicly, so supporting data is not available.

## Abbreviations

ART: HIV antiretroviral therapy
Endo: endocarditis
HCV: hepatitis C virus
HIV: human immunodeficiency virus
ID: infectious diseases
IQR: inter-quartile range
IV: intravenous
LOS: length of stay
MDMA: 3,4-methylenedioxy-methamphetamine
osteo: osteomyelitis
OUD: opioid use disorder
PDD: patient-directed discharge
PrEP: HIV pre-exposure prophylaxis
PWID: people who injection drugs
SA: septic arthritis
SIRI: severe injection-related infection
SSTI: skin and soft tissue infection
SUD: substance use disorder
USD: United States dollars

## References

[1] Wurcel AG, Anderson JE, Chui KK, et al. Increasing infectious endocarditis admissions among young people who inject drugs. Open Forum Infect Dis. 2016;3(3):ofw157. 27800528 10.1093/ofid/ofw157 PMC5084714

[2] Shiels MS, Freedman ND, Thomas D, et al. Trends in U.S. drug overdose deaths in non-Hispanic black, Hispanic, and non-Hispanic white persons, 2000–2015. Ann Intern Med. 2018;168(6):453–455. 29204603 10.7326/M17-1812 PMC6309971

[3] Goodnough A. Injecting drugs can ruin a heart. How many second chances should a user get? *New York Times*; 2018.

[4] McCarthy NL, Baggs J, See I, et al. Bacterial infections associated with substance use disorders, large cohort of United States hospitals, 2012–2017. Clin Infect Dis. 2020. 10.1093/cid/ciaa008 PMC7900878 31907515

[5] Wong CY, Zhu W, Aurigemma GP, et al. Infective endocarditis among persons aged 18–64 years with HIV, hepatitis C infection, or opioid use disorder – United States, 2007–2017. Clin Infect Dis. 2020. 10.1093/cid/ciaa372 32270861

[6] Schranz AJ, Fleischauer A, Chu VH, et al. Trends in drug use-associated infective endocarditis and heart valve surgery, 2007 to 2017: a study of statewide discharge data. Ann Intern Med. 2019;170(1):31–40. 30508432 10.7326/M18-2124 PMC6548681

[7] Leahey PA, LaSalvia MT, Rosenthal ES, et al. High morbidity and mortality among patients with sentinel admission for injection drug use-related infective endocarditis. Open Forum Infect Dis. 2019;6(4):ofz089. 30949535 10.1093/ofid/ofz089 PMC6441563

[8] Straw S, Baig MW, Gillott R, et al. Long-term outcomes are poor in intravenous drug users following infective endocarditis, even after surgery. Clin Infect Dis. 2019. 10.1093/cid/ciz869 31504326

[9] Barocas JA, Eftekhari Yazdi G, Savinkina A, et al. Long-term infective endocarditis mortality associated with injection opioid use in the United States: a modeling study. Clin Infect Dis. 2020. 10.1093/cid/ciaa1346 PMC8662770 32901815

[10] Marks LR, Munigala S, Warren DK, et al. A comparison of medication for opioid use disorder treatment strategies for persons who inject drugs with invasive bacterial and fungal infections. J Infect Dis. 2020;222(Suppl 5):S513–S520. 32877547 10.1093/infdis/jiz516 PMC7566615

[11] Jo Y, Nosal R, Vittori A, et al. Effect of initiation of medications for opioid use disorder on hospitalization outcomes for endocarditis and osteomyelitis in a large private hospital system in the United States, 2014. Addiction. 2021;116(8):2127–2134. 33394516 10.1111/add.15393 PMC8359423

[12] Fanucchi LC, Walsh SL, Thornton AC, et al. Outpatient parenteral antimicrobial therapy plus buprenorphine for opioid use disorder and severe injection-related infections. Clin Infect Dis. 2019. 10.1093/cid/ciz654 PMC7931831 31342057

[13] Nolan NS, Marks LR, Liang SY, et al. Medications for opioid use disorder associated with less against medical advice discharge among persons who inject drugs hospitalized with an invasive infection. J Addict Med. 2020. 10.1097/ADM.0000000000000725 PMC7995266 32804690

[14] Marks LR, Liang SY, Muthulingam D, et al. Evaluation of partial oral antibiotic treatment for persons who inject drugs and are hospitalized with invasive infections. Clin Infect Dis. 2020. 10.1093/cid/ciaa365 PMC7745005 32239136

[15] Marks LR, Munigala S, Warren DK, et al. Addiction medicine consultations reduce readmission rates for patients with serious infections from opioid use disorder. Clin Infect Dis. 2019;68(11):1935–1937. 30357363 10.1093/cid/ciy924 PMC6522678

[16] Englander H, Dobbertin K, Lind BK, et al. Inpatient addiction medicine consultation and post-hospital substance use disorder treatment engagement: a propensity-matched analysis. J Gen Intern Med. 2019;34(12):2796–2803. 31410816 10.1007/s11606-019-05251-9 PMC6854181

[17] Larochelle MR, Bernson D, Land T, et al. Medication for opioid use disorder after nonfatal opioid overdose and association with mortality: a cohort study. Ann Intern Med. 2018;169(3):137–145. 29913516 10.7326/M17-3107 PMC6387681

[18] Serota DP, Bartholomew TS, Tookes HE. Evaluating differences in opioid and stimulant use-associated infectious disease hospitalizations in Florida, 2016–2017. Clin Infect Dis. 2020. 10.1093/cid/ciaa1278 PMC8492144 32886747

[19] National Academies of Sciences, Engineering, and Medicine. Opportunities to improve opioid use disorder and infectious disease services: integrating responses to a dual epidemic; 2020. 32293827

[20] Serota DP, Niehaus ED, Schechter MC, et al. Disparity in quality of infectious disease vs addiction care among patients with injection drug use-associated *Staphylococcus aureus* bacteremia. Open Forum Infect Dis. 2019;6(7):ofz289. 31304193 10.1093/ofid/ofz289 PMC6612813

[21] Serota DP, Barocas JA, Springer SA. Infectious complications of addiction: a call for a new subspecialty within infectious diseases. Clin Infect Dis. 2020;70(5):968–972. 31420651 10.1093/cid/ciz804 PMC7319263

[22] Serota DP, Chueng TA, Schechter MC. Applying the infectious diseases literature to people who inject drugs. Infect Dis Clin North Am. 2020;34(3):539–558. 32782101 10.1016/j.idc.2020.06.010 PMC8164212

[23] Serota DP, Vettese T. New answers for old questions in the treatment of severe infections from injection drug use. J Hosp Med. 2020;15(10):606–612. 31869292 10.12788/jhm.3342

[24] Serota DP, Kraft CS, Weimer MB. Treating the symptom but not the underlying disease in infective endocarditis: a teachable moment. JAMA Intern Med. 2017;177(7):1026–1027. 28505250 10.1001/jamainternmed.2017.1489

[25] Tookes H, Diaz C, Li H, et al. A cost analysis of hospitalizations for infections related to injection drug use at a county safety-net hospital in Miami, Florida. PLOS One. 2015;10(6):e0129360. 26075888 10.1371/journal.pone.0129360 PMC4468183

[26] Coye AE, Bornstein KJ, Bartholomew TS, et al. Hospital costs of injection drug use in Florida. Clin Infect Dis. 2021;72(3):499–502. 32564077 10.1093/cid/ciaa823 PMC8489420

[27] Levine H, Bartholomew TS, Rea-Wilson V, et al. Syringe disposal among people who inject drugs before and after the implementation of a syringe services program. Drug Alcohol Depend. 2019;202:13–17. 31280002 10.1016/j.drugalcdep.2019.04.025 PMC6854527

[28] Bornstein KJ, Coye AE, St Onge JE, et al. Hospital admissions among people who inject opioids following syringe services program implementation. Harm Reduct J. 2020;17(1):30. 32398059 10.1186/s12954-020-00376-1 PMC7216361

[29] Ginoza MEC, Tomita-Barber J, Onugha J, et al. Student-run free clinic at a syringe services program, Miami, Florida, 2017–2019. Am J Public Health. 2020;110(7):988–990. 34166061 10.2105/AJPH.2020.305705 PMC7287550

[30] Eaton EF. We thought we created a safety net. We were wrong. Clin Infect Dis. 2020. 10.1093/cid/ciaa1291 PMC8492159 32881991

[31] Damschroder LJ, Aron DC, Keith RE, Kirsh SR, et al. Fostering implementation of health services research findings into practice: a consolidated framework for advancing implementation science. Implement Sci. 2009;4:50. 19664226 10.1186/1748-5908-4-50 PMC2736161

[32] Englander H, Wilson T, Collins D, et al. Lessons learned from the implementation of a medically enhanced residential treatment (MERT) model integrating intravenous antibiotics and residential addiction treatment. Subst Abus. 2018;39(2):225–232. 29595367 10.1080/08897077.2018.1452326 PMC6519053

[33] Fanucchi LC, Lofwall MR, Nuzzo PA, et al. In-hospital illicit drug use, substance use disorders, and acceptance of residential treatment in a prospective pilot needs assessment of hospitalized adults with severe infections from injecting drugs. J Subst Abuse Treat. 2018;92:64–69. 30032946 10.1016/j.jsat.2018.06.011

[34] Fanucchi LC, Walsh SL, Thornton AC, et al. Integrated outpatient treatment of opioid use disorder and injection-related infections: a description of a new care model. Prev Med. 2019;128:105760. 31251946 10.1016/j.ypmed.2019.105760

[35] Blevins SR, Stivers T, Sabitus K, et al. 83. A descriptive analysis of a multi-disciplinary approach to opioid use disorder treatment within an infectious diseases clinic. Open Forum Infect Dis. 2020;7(Supplement_1):S173.

[36] Rosenthal ES, Silk R, Mathur P, et al. Concurrent initiation of hepatitis C and opioid use disorder treatment in people who inject drugs. Clin Infect Dis. 2020. 10.1093/cid/ciaa105 PMC7755091 32009165

[37] Barocas JA, Gai MJ, Amuchi B, Jawa R, et al. Impact of medications for opioid use disorder among persons hospitalized for drug use-associated skin and soft tissue infections. Drug Alcohol Depend. 2020;215:108207. 32795883 10.1016/j.drugalcdep.2020.108207 PMC7502512

[38] Barocas JA, Morgan JR, Wang J, et al. Outcomes associated with medications for opioid use disorder among persons hospitalized for infective endocarditis. Clin Infect Dis. 2020. 10.1093/cid/ciaa062 PMC7850516 31960025

[39] Kimmel SD, Walley AY, Li Y, et al. Association of treatment with medications for opioid use disorder with mortality after hospitalization for injection drug use-associated infective endocarditis. JAMA Netw Open. 2020;3(10):e2016228. 33052402 10.1001/jamanetworkopen.2020.16228 PMC7557514

[40] Metsch LR, Feaster DJ, Gooden L, et al. Effect of patient navigation with or without financial incentives on viral suppression among hospitalized patients with HIV infection and substance use: a randomized clinical trial. JAMA. 2016;316(2):156–170. 27404184 10.1001/jama.2016.8914 PMC5339876

[41] Kimmel SD, Rosenmoss S, Bearnot B, et al. Rejection of patients with opioid use disorder referred for post-acute medical care before and after an anti-discrimination settlement in Massachusetts. J Addict Med. 2021;15(1):20–26. 32675798 10.1097/ADM.0000000000000693 PMC7859880

[42] Castillo M, Ginoza MEC, Bartholomew TS, et al. When is an abscess more than an abscess? Syringe services programs and the harm reduction safety-net: a case report. Harm Reduct J. 2020;17(1):34. 32487084 10.1186/s12954-020-00381-4 PMC7268493

