# Supplementary material for: Harm reduction for the treatment of patients with severe injection-related infections: description of the Jackson SIRI Team
Source: Ann Med. 2021 Nov 2;53(1):1960–8. doi: 10.1080/07853890.2021.1993326 (PMC8567885; doi:10.1080/07853890.2021.1993326)
Supplement: Supplemental Material [file IANN_A_1993326_SM5103.zip › Appendix_2.docx]

**Severe Injection-Related Infection (SIRI) Team Consult Note**

Name: _
MRN: _
Date of birth: _
Date of admission: _

Primary team: _

Consult request: evaluation and treatment of injection-related infection

History of Present Illness:
_

DSM 5 Criteria - Substance: _

| Preset? | In the last year have you: |
| --- | --- |
| _ | 1. using more or longer than intended |
| _ | 2. tried to cut down but couldn’t |
| _ | 3. lots of time spent using or recovering from drug use |
| _ | 4. wanted to use so badly you couldn't think of anything else |
| _ | 5. found that using or being sick from using often interfered with taking care of your home or family? Or caused job troubles? Or school problems? |
| _ | 6. continued to use even though it was causing trouble with your family or friends? |
| _ | 7. given up important or enjoyable activities in favor of drug use |
| _ | 8. use in hazardous situations |
| _ | 9. use despite it causing depression, anxiety, or medical problems |
| _ | 10. tolerance |
| _ | 11. withdrawal |
| _ | Total: Mild 2-3; Moderate 4-5; Severe ≥6 |

Substance Use History:
· Current opioid use: _
· Other substances used: _
· Alcohol: _
· Tobacco: _
· Cannabis: _
· Which substance injected: _
· Past hx of opioid treatment (including suboxone/methadone/detox): _
· Past hx of opioid overdose: _
· Past hx of medical complications from drug use: _
· Last use (substance and timing): _
· PDMP report: _

Social History:
· Housing: _
· Occupation: _
· Education: _
· Relationships: _

· Sexual activity: _
· Supportive people in life: _

Review of Systems:
_

Pertinent Medical/Surgical History:
_

Fam Hx of addiction:
_

Allergies:
_

Medication List:
_

Vitals:

_

Physical Exam:
_

Labs/Path:
_

Microbiology:
_

Imaging:
_

Assessment and Recommendations:
_

RECOMMENDATIONS:
_

==============================================
SIRI team quality measures
*Screening tests (to be done now and then at least annually):*
o HIV: _

o HAV (total Ab): _
o HBV: _
o HCV: _
o Syphilis: _
o Gonorrhea/Chlamydia: _
o TB: _

*Vaccinations –please administer during hospitalization*
o Hepatitis A vaccine: _
o Hepatitis B vaccine: _
o PPV-23 vaccine:_
o Tdap: _

*Medications:*
o PrEP: _
o ART: _
o Medications for opioid use disorder: _

*Follow-up/Discharge*
o If patient desires to leave AMA:
 o Call SIRI phone ASAP at 305-XXX-XXXX
 o AMA antibiotic contingency plan: _
 o Order antibiotic to JMH pharmacy, call 305-585-XXXX
o Patients will OUD are provided Naloxone (narcan) at bedside by SIRI team
o Referred patient to IDEA SSP with card including address, phone, hours
o Counseled patient prior to discharge on safer injection practices
